# Relationship Between Circulating MicroRNAs and Left Ventricular Hypertrophy in Hypertensive Patients

**DOI:** 10.3389/fcvm.2022.798954

**Published:** 2022-04-13

**Authors:** Elisangela C. P. Lopes, Layde R. Paim, Luís F. R. S. Carvalho-Romano, Edmilson R. Marques, Eduarda O. Z. Minin, Camila F. L. Vegian, José A. Pio-Magalhães, Lício A. Velloso, Otavio R. Coelho-Filho, Andrei C. Sposito, José R. Matos-Souza, Wilson Nadruz, Roberto Schreiber

**Affiliations:** Department of Internal Medicine, School of Medical Sciences, State University of Campinas, São Paulo, Brazil

**Keywords:** hypertension, left ventricular hypertrophy, cardiac hypertrophy, microRNAs, miR-145-5p

## Abstract

**Objective:**

Left ventricular hypertrophy (LVH) is a common complication of hypertension and microRNAs (miRNAs) are considered to play an important role in cardiac hypertrophy development. This study evaluated the relationship between circulating miRNAs and LVH in hypertensive patients.

**Methods:**

Two cohorts [exploratory (*n* = 42) and validation (*n* = 297)] of hypertensive patients were evaluated by clinical, laboratory and echocardiography analysis. The serum expression of 754 miRNAs in the exploratory cohort and 6 miRNAs in the validation cohort was evaluated by the TaqMan OpenArray^®^ system and quantitative polymerase chain reaction, respectively.

**Results:**

Among the 754 analyzed miRNAs, ten miRNAs (miR-30a-5p, miR-let7c, miR-92a, miR-451, miR-145-5p, miR-185, miR-338, miR-296, miR-375, and miR-10) had differential expression between individuals with and without LVH in the exploratory cohort. Results of multivariable regression analysis adjusted for confounding variables showed that three miRNAs (miR-145-5p, miR-451, and miR-let7c) were independently associated with LVH and left ventricular mass index in the validation cohort. Functional enrichment analysis demonstrated that these three miRNAs can regulate various genes and pathways related to cardiac remodeling. Furthermore, *in vitr*o experiments using cardiac myocytes demonstrated that miR-145-5p mimic transfection up-regulated the expression of brain and atrial natriuretic peptide genes, which are markers of cardiac hypertrophy, while anti-miR-145-5p transfection abrogated the expression of these genes in response to norepinephrine stimulus.

**Conclusions:**

Our data demonstrated that circulating levels of several miRNAs, in particular miR-145-5p, miR-451, and let7c, were associated with LVH in hypertensive patients, indicating that these miRNAS may be potential circulating biomarkers or involved in hypertension-induced LV remodeling.

## Introduction

Hypertension is characterized by high blood pressure (BP) levels and is associated with target-organs damage and increased risk of cardiovascular events (1–3). Sustained BP elevation increases left ventricular (LV) wall tension, and may commonly lead to a compensatory increase in LV mass (LVM), known as left ventricular hypertrophy (LVH). Importantly, LVH is associated with an increased risk of cardiovascular morbidity and mortality (4, 5).

Alternative factors, including genetic (6), epigenetic (7) and environmental (8) factors, can contribute to LVH. MicroRNAs (miRNAs) are a class of small non-coding RNAs that modulate gene expression at the post-transcriptional level. They are believed to comprise about 1% to 5% of the human genome, and currently at least 2,654 mature miRNA sequences have been described (9). Several studies in humans and experimental models have suggested an important role of miRNAs in cardiac hypertrophy (10), but little is known regarding the impact of miRNAs on hypertension-induced LV remodeling. In this regard, an association between selected circulating miRNAs and LVM has been reported in hypertensive individuals (11–15).

This study aimed at identifying circulating miRNAs related to LVH in hypertensive individuals and evaluating the functional role of selected miRNAs on markers of cardiac myocyte hypertrophy *in vitro*.

## Materials and Methods

Additional detailed methods are included in the Supplementary Data.

The authors declare that all supporting data are available within the article (and in the Data Supplement).

### Study Populations

The present study included two cohorts of consecutive hypertensive patients followed at the Hypertension Outpatient Clinic of the Clinics Hospital of the University of Campinas who were enrolled from 2018 to 2019. The exploratory cohort included 42 patients (26 with LVH and 16 without LVH) and the validation cohort comprised 297 patients (162 with LVH and 135 without LVH). Exclusion criteria were age under 18 years, identifiable causes of secondary hypertension, evidence of significant cardiac valve disease and hypertrophic cardiomyopathy. The research was carried out in accordance with the Declaration of Helsinki. This study was approved by the Human Research Ethics Committee of the University of Campinas, and all patients gave written informed consent to participate.

### Clinical, Laboratory, and Echocardiography Data

Clinical data were gathered from each participant and included information on: age, sex, smoking, hypertension, diabetes mellitus, use of antihypertensive medications, body mass index (BMI), BP, heart rate. BP and heart rate were measured in the sitting position using a validated digital oscillometric device (HEM-705CP; Omron Healthcare, Kyoto, Japan) with appropriate cuff sizes. BMI was calculated as body weight divided by height squared (kg/m^2^). Fasting low density lipoprotein (LDL)-cholesterol, high-density lipoprotein (HDL)-cholesterol, triglycerides, creatinine, and glucose levels were measured using standard laboratory techniques. Hypertension was defined as systolic BP ≥140 mmHg or diastolic ≥90 mmHg or use of antihypertensive medications. Diabetes mellitus was diagnosed if fasting blood glucose was ≥126 mg/dl or when participants were taking hypoglycemic medications.

Echocardiography was performed by a single physician using a Vivid q device (General Electric, Milwaukee, Wisconsin, USA) equipped with a 3S-RS transducer, as previously described (16–18). LVMI was calculated as LV mass/body surface area. Relative wall thickness (RWT) was measured as 2^*^posterior wall thickness/LV diastolic diameter. LVH was defined as LVMI ≥95 g/m^2^ and ≥ 115 g/m^2^ in women and men, respectively. LV geometric patterns were defined as follows: normal geometry (No LVH and RWT ≤0.42), concentric remodeling (No LVH and RWT >0.42), eccentric hypertrophy (LVH and RWT ≤0.42) and concentric hypertrophy (LVH and RWT >0.42). LV ejection fraction was estimated by the Simpson's method.

### Extraction and Analysis of Serum miRNA Expression

Samples from both cohorts were extracted using the miRNeasy Serum/Plasma Kit (Qiagen). The quality of miRNA was assessed by measuring the percentage of miRNAs in the amount of small RNA using a Bioanalyzer 2,100 (Agilent, Santa Clara, CA), as previously reported (19). In the exploratory cohort, the miRNA profile was analyzed by the TaqMan OpenArray Human MicroRNA system, a quantitative polymerase chain reaction (qPCR)-based miRNA array platform that contains 754 microRNAs on a microfluidic platform across two sets of primer pools, panel A and B (LifeTechnologies). Data were normalized using the global normalization method as suggested by the manufacturer and previous reports (20). Six circulating miRNAs with the highest fold change in the exploratory study were chosen for the validation in the validation cohort by qRT-PCR using a customized plate (LifeTechnologies). Reverse transcription was performed using the SuperScript^®^III First Strand Synthesis Kit. Specific miRNA PCR primers were synthesized by Life Technologies. Real-time PCR assays were performed with the TaqMan Master Mix (Life Technologies) on a 7900HT FAST Real- Time PCR System (Life Technologies). The comparative Ct (ΔΔCt) method to quantify relative gene expression was used, and fold change (FC) was calculated as FC = 2- ^ΔΔCt^, where Ct is defined as the PCR cycle number at which the fluorescence meets the threshold in the amplification plot (21). Data were normalized using a geometric mean of U6 snRNA (non-coding small nuclear RNA) and miR-16 as the housekeeping genes.

### Gene Set Enrichment Analysis

To understand the biological relevance of differentially expressed miRNAs, we performed functional enrichment analysis. The miRNAs differentially expressed between patients with and without LVH that significantly correlated with LVMI were uploaded into miRWalk (version 2) (22). To strengthen the data, only mRNAs predicted in at least four of five tools (miRanda, miRDB, miRWalk, RNA22, and TargetScan) were considered as possible miRNA targets. We used the Database for Annotation, Visualization, and Integrated Discovery (DAVID) to determine the enriched pathways. Only pathways with more than 10 genes, fold enrichment >1.5, and Fisher's exact test *p*-value < 0.05 were considered (23).

### HL-1 Cells Culture and miRNA Transfection

We used the adult mouse atrial muscle cell line HL-1, which has been extensively used to assess mechanisms related to cardiac myocyte hypertrophy (24, 25), to evaluate the functional impact of selected miRNAs. HL-1 cells were cultured at 37°C and 5% CO_2_ atmosphere in Claycomb medium (Sigma, 51800C) supplemented with 2 mM L-Glutamine (Sigma, G7513), penicillin-streptomycin (Sigma, P4333) and 10 %(v/v) FCS (Sigma; F2442) in the absence or presence of 100 μM of norepinephrine (Sigma, A0937) as a hypertrophic stimulus (26, 27) for 2 weeks (28) on gelatin-fibronectin [0.02 % (w/v) gelatin; 5 mg/ml fibronectin] (Sigma, F1141) pre-coated plates (29). Then, HL-1 cells were transiently transfected with miR-145-5p mimetic, anti-miR-145-5p or negative control miRNA (Applied Biosystems, ThermoFisher Scientific, Grand Island, NY) at final concentrations of 30 nM. This assay was carried out using the Lipofectamine 2000 Reagent (Invitrogen, Carlsbad, CA) following the manufacturer's instructions. Cells were harvested for subsequent analyses 48 h after the transfection. All transfection experiments were carried out in triplicate.

### Real-Time Quantitative PCR Analysis From Cells Culture

For detecting the *in vitro* expression of miRNAs and markers of cardiac myocyte hypertrophy, total RNA from cardiac myocytes was isolated using Mirvana Paris Kit (Applied Biosystems, CA). We evaluated the expression of brain natriuretic peptide (Nppb) and atrial natriuretic peptide (Nppa) genes, which are markers of cardiac myocyte hypertrophy, and miR-145-5p using the TaqMan Gene Expression Assays Kit (Applied Biosystems, CA). The polymerase chain reaction was performed in a StepOnePlus ™ System (ThermoFisher) real-time PCR system. Gapdh (Mm99999915_g1) was used as endogenous control for Nppb and Nppa expression, while U6 snRNA was used to normalize miR-145-5p expression.

### Statistical Analysis

Continuous variables with normal or non-normal distribution are presented as mean ± standard deviation (SD) or median [25th, 75th percentiles]. Differences in continuous variables with normal or non-normal distribution between the studied groups (with and without LVH) were evaluated by unpaired student's *t*-test and Mann-Whitney U-test, respectively. Chi-square test was used to compare categorical variables. Differences in continuous variables derived from cell assays were evaluated by one-way analysis of variance (ANOVA) followed by the Tukey test. The correlation between echocardiographic variables and log-transformed expression of miRNAs was assessed by the Person's Method. Multivariable linear regression analysis evaluated the association of log-transformed expression of circulating miRNAs with LVH, LVMI, and LV geometric patterns in the validation cohort, adjusting for variables that have been reported to influence LV remodeling: age, sex, diabetes, body mass index, systolic blood pressure, creatinine, smoking, and antihypertensive classes (5). *p*-value < 0.05 was considered statistically significant. SPSS 15.0 software was used for statistical analyses.

## Results

### Clinical Characteristics of Participants

The characteristics of the exploratory cohort (*n* = 42; 57.7 ± 8.5 years, 52% males) and the validation cohort (*n* = 297; 61.3 ± 12.1 years, 43% males) are shown in the Supplementary Table S1. The cohorts had similar characteristics except for higher BP and rates of diabetes and use of diuretics, greater LV wall thickness and relative wall thickness values, and lower LDL-cholesterol levels in the validation cohort.

Clinical, laboratory, and echocardiographic features of the exploratory and validation cohorts according to the presence or not of LVH are presented in Table 1. There were no differences in clinical and laboratory characteristics between participants according to LVH status, except for greater systolic BP and creatinine levels in LVH patients of the validation cohort compared with those without LVH. In addition, patients with LVH had greater LV dimensions, LV mass and RWT than those without LVH in both cohorts.

**Table 1:** Clinical, laboratory and echocardiographic characteristics of the cohorts.

**Characteristics**	**Exploratory cohort**			**Validation cohort**		
	**No LVH (*n* = 16)**	**LVH (*n* = 26)**	***p***	**No LVH (*n* = 135)**	**LVH (*n* = 162)**	***p***
Age, years	56.5 ± 8.2	58.4 ± 8.8	0.49	59.9 ± 12.9	62.4 ± 11.3	0.09
Sex (Male/Female)	7/9	15/11	0.38	62/73	66/96	0.37
Smokers, (%)	2 (12)	6 (23)	0.66	16 (12)	17 (11)	0.85
Diabetics, (%)	4 (25)	13 (50)	0.21	76 (56)	98 (60)	0.54
Body mass index, kg/m^2^	28.9 ± 4.6	29.8 ± 4.6	0.53	30.0 ± 6.0	30.4 ± 5.4	0.54
LDL-cholesterol, mg/dL	105 ± 40	117 ± 29	0.30	91 ± 33	94 ± 33	0.40
HDL-cholesterol, mg/dL	48 ± 14	48 ± 11	0.93	46 ± 12	46 ± 13	0.95
Triglycerides, mg/dL	110 [78, 181]	139 [94, 212]	0.21	123 [86, 180]	124 [90, 175]	0.94
Glucose, mg/dL	99 [86, 114]	100 [92, 137]	0.40	101 [91, 117]	102 [90, 130]	0.73
Creatinine, mg/dL	0.90 [0.76, 0.94]	0.89 [0.74, 1.07]	0.86	0.88 [0.73, 1.07]	0.99 [0.76, 1.20]	0.030
Systolic blood pressure, mm Hg	137.1 ± 21.1	142.3 ± 19.5	0.42	144.8 ± 23.9	152.3 ± 25.4	0.010
Diastolic blood pressure, mm Hg	73.5 ± 11.1	78.4 ± 14.3	0.25	82.2 ± 15.1	84.4 ± 15.3	0.23
Diuretics, *n* (%)	12 (75)	20 (77)	0.88	66 (49)	90 (55)	0.30
CCB, *n* (%)	9 (56)	13 (50)	0.94	54 (40)	78 (48)	0.20
β-Blockers, *n* (%)	8 (50)	13 (50)	0.99	59 (44)	70 (43)	0.93
ACEI or ARB, *n* (%)	12 (75)	21 (81)	0.95	118 (87)	143 (88)	0.96
Interventricular septum thickness, mm	8.9 ± 0.6	10.7 ± 1.3	<0.001	9.4 ± 1.2	11.4 ± 1.6	<0.001
Posterior wall thickness, mm	8.9 ± 0.6	10.6 ± 1.1	<0.001	9.5 ± 1.2	11.3 ± 1.3	<0.001
LV end-diastolic diameter, mm	47.6 ± 2.5	51.0 ± 5.3	0.026	47.0 ± 4.4	51.9 ± 6.4	<0.001
LV ejection fraction, %	68.9 ± 5.8	65.1 ± 4.4	0.043	67.5 ± 6.8	63.2 ± 10.9	<0.001
Relative wall thickness, mm	0.37 ± 0.03	0.42 ± 0.01	0.004	0.41 ± 0.06	0.44 ± 0.07	<0.001
LV mass index, g/m^2^	89.6 ± 6.3	138.8 ± 4.7	<0.001	85.7 ± 13.7	133.1 ± 28.7	<0.001

*Continuous data with normal and non-normal distribution are presented as mean ± standard deviation and median [25th, 75th percentiles]. ACEI or ARB, angiotensin-converting enzyme inhibitors or angiotensin receptor blockers; CCB, calcium channel blockers; HDL, high density lipoprotein; LDL, low density lipoprotein; LV, left ventricular; LVH, left ventricular hypertrophy*.

### MicroRNA Expression Levels and LV Remodeling in Exploratory and Validation Cohorts

**Figure 1:**
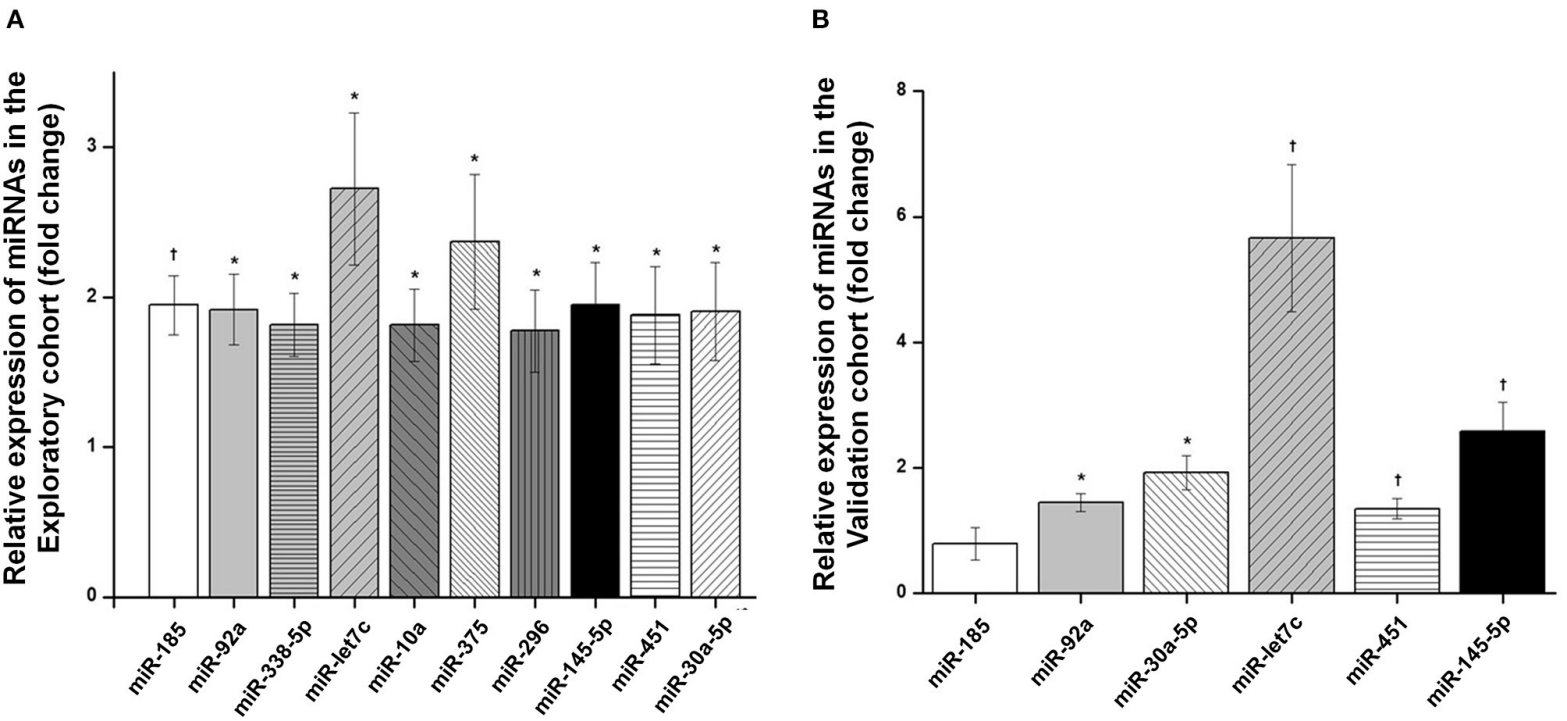
Differential expression analysis of miRNAs obtained in serum of hypertensive patients. **(A)** Differentially expressed serum miRNAs (fold change) in patients with left ventricular hypertrophy (LVH) compared with patients without LVH in the exploratory cohort. **(B)** Differentially expressed serum miRNAs (fold change) in patients with LVH compared with patients without LVH in the validation cohort. Box and whisker plots are represented for each miRNA and indicate the fold change in expression of LVH patients compared with patients without LVH. p-values from independent Mann-Whitney test are presented. †*p* < 0.001; *p < 0.05.

The analysis of serum expression of 754 miRNAs using the OpenArray MicroRNA System in the exploratory cohort showed that 357 miRNAs were expressed in at least one of the two groups (with and without LVH). For the differential analysis, only 122 miRNAS that were expressed in at least 50% of the participants within each group were considered (Supplementary Table S2). Of these, 10 miRNAs (miR-30a-5p, miR-let7c, miR-92a, miR-451, miR-145-5p, miR-185, miR-338, miR-296, miR-375 and miR-10) were significantly upregulated in patients with LVH compared with those without LVH (Figure 1A, Supplementary Table S3 and Figure S1). Of these 10 miRNAs, six miRNAs with the highest fold change in the exploratory study (miR-30a-5p, miR-let7c, miR-92a, miR-451, miR-145-5p, miR-185) were chosen to have their serum expression measured by qRT-PCR in the validation cohort. All except one miRNA (miR-185) showed higher expression in LVH patients when compared with those without LVH in the validation cohort (Figure 1B and Supplementary Table S3). Bivariate correlation analysis was then performed to evaluate the relationship of circulating miRNAs with LVMI in the validation cohort. Of the six tested miRNAs, miR-145-5p (*r* = 0.153; *p* = 0.011), miR-451 (*r* = 0.162; *p* = 0.016) and miR-let7c (*r* = 0.242; *p* < 0.001) significantly correlated with LVMI, while miR-92a (*r* = 0.098; *p* = 0.10), miR30a-5p (*r* = 0.093; *p* = 0.12) and miR-185 (*r* = −0.155; *p* = 0.07) did not. Systolic BP was positively correlated with miR-30a-5p (*r* = 0.146; *p* = 0.015) and miR-451 (*r* = 0.213; *p* = 0.002), while no correlation between the studied miRNAs and the presence of diabetes was observed (Supplemental Table S4).

Results of multivariable linear regression analyses adjusted for potentially relevant confounders (age, sex, body mass index, systolic blood pressure, diabetes mellitus, creatinine, smoking, and antihypertensive medications) showed that miR-30a-5p, miR-let7c, miR-92a, miR-451, miR-145-5p remained associated with LVH, while only miR-145-5p, miR-let7c, and miR-451 showed an independent association with LVMI in the validation cohort (Supplementary Table S5). As a secondary analysis, we evaluated the relationship between LV geometric patterns and selected miRNAs (miR-30a-5p, miR-let7c, miR-92a, miR-451, miR-145-5p, miR-185) adjusted for potential confounders in the validation cohort. Compared with the normal geometric pattern, patients with concentric hypertrophy had greater expression of miR-let7c, miR-92a, miR-145-5p, miR-30a-5p, and miR-451, while patients with eccentric hypertrophy had greater expression of miR-let7c and miR-145-5p (Supplementary Table S6).

### Gene Set Enrichment Analysis

**Figure 2:**
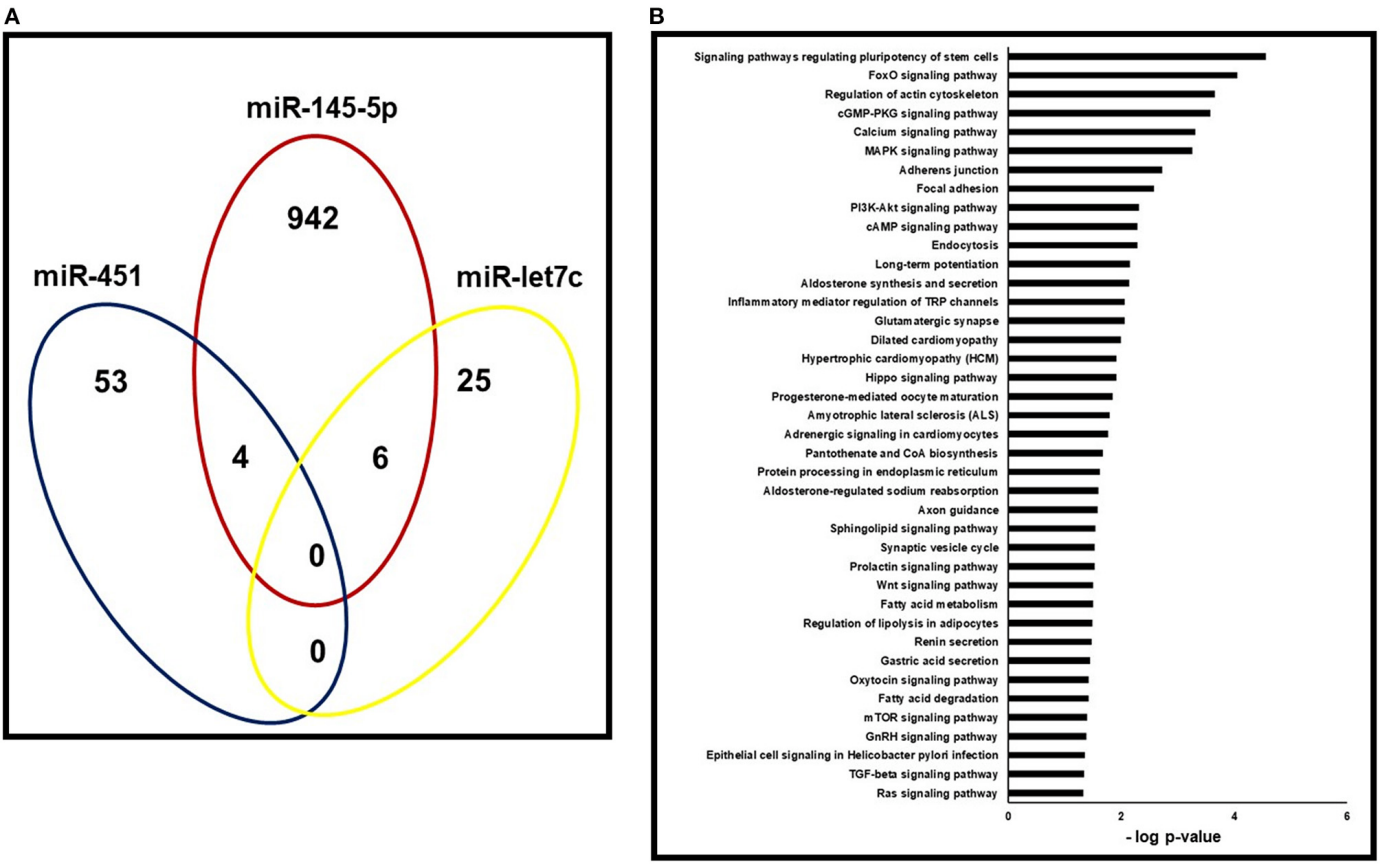
Pathway enrichment analysis of predicted target genes. **(A)** Gene ontology analysis for the three miRNAs (miR-145-5p, miR-451 and miR-let7c) that correlated with both LVH and LVMI in the validation cohort. Venn's diagram of genes targeted by each of the three miRNAs with *p* < 0.05. **(B)**- Gene set enrichment analyses of pathways targeted by miR-145-5p, miR-451 and miR-let7c.

Using the miRWALK2.0 software, 1030 predicted genes were identified to be targeted by the three miRNAs that were associated with both LVH and LVMI (miR-145-5p, miR-let7c and miR-451). The number of genes targeted by each of the miRNAs is shown in the Venn diagram presented in Figure 2A. Figure 2B shows the predicted pathways of the aforementioned miRNAs. Pathways related to cardiac remodeling and vascular diseases, and metabolic and inflammatory pathways were predicted, including calcium and adrenergic signaling pathways, focal adhesion and hypertrophic cardiomyopathy.

### Overexpression of miR-145-5p Increases Hypertrophic Genes

**Figure 3:**
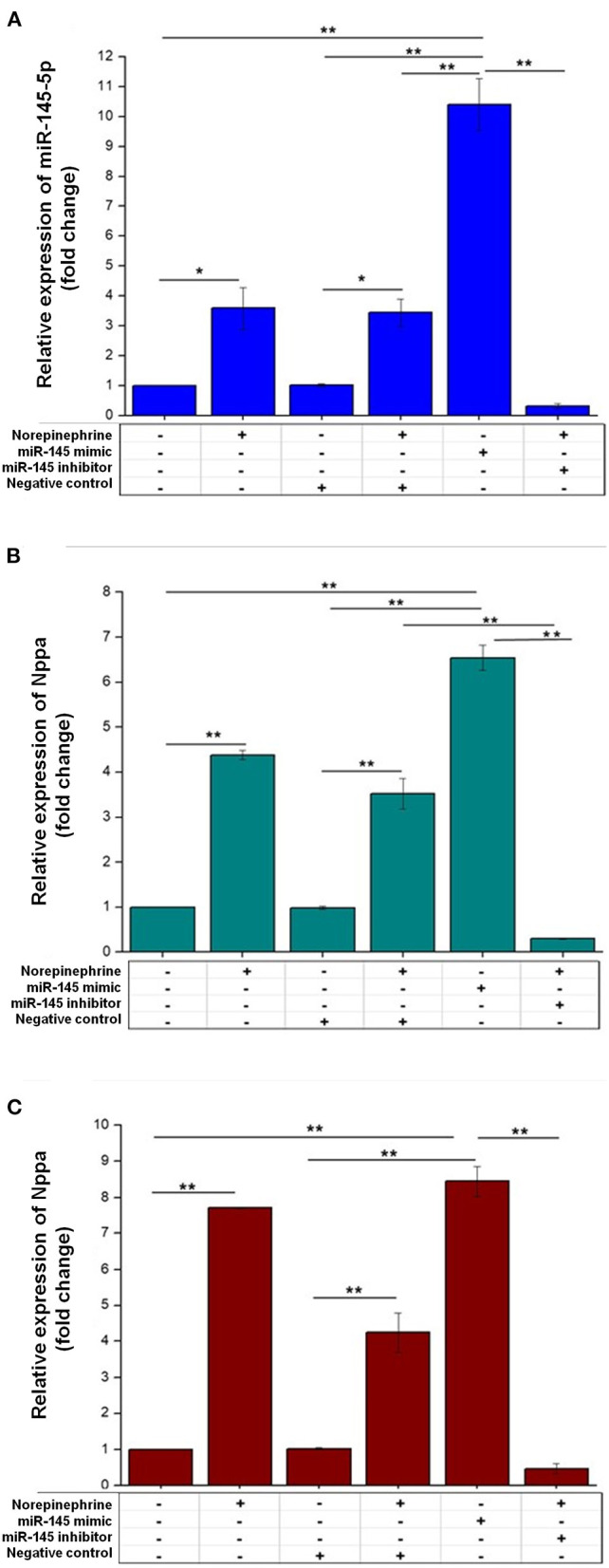
Effects of miR-145-5p on cardiac myocyte hypertrophy. **(A)** The mRNA levels of miR-145-5p were increased in HL-1 cells treated with norepinephrine or transfected with miR-145-5p mimic (30 nM). Relative miR-145-5p expression corresponded to average expressions (fold change) normalized to U6. **(B,C)** HL-1 cells transfected with miR-145-5p mimic (30 nM) increased the mRNA level of Nppa (atrial natriuretic peptide gene) and Nppb (brain natriuretic peptide gene), while transfection with miR-145-5p inhibitor (30 nM) abrogated norepinephrine-induced increases in Nppa and Nppb. Relative expressions of hypertrophy markers were expressed as the average expressions (fold change) normalized to Glyceraldehyde-3-posphate dehydrogenase (GAPDH), by RT-PCR analysis. Data are presented as mean ± SEM (*n* = 3). Differences in the expression of Nppa, Nppb and miR-145 were assessed by one-way ANOVA and Tukey *post hoc* test. *p < 0.05; **p < 0.001.

Due to controversial results in the literature (30–32), we decided to assess whether miR-145 is involved in the regulation of cardiac myocyte hypertrophy *in vitro*. We first evaluated the expression of this miRNA in HL-1-cells. We found that miR-145-5p was constitutively expressed in these cells and that its expression increased by ≈ 3-fold after norepinephrine stimulus and by ≈10-fold after transfection with miR-145-5p mimic (Figure 3A). The expression of Nppa and Nppb significantly increased in HL-1-cells transfected with miR-145-5p mimic (30nM) and in response to norepinephrine (Figures 3B,C). By contrast, transfection of anti-miR-145-5p (30nM) markedly decreased norepinephrine-induced expression of Nppa and Nppb when compared with cells transfected with negative control (Figures 3B,C).

## Discussion

In the present study, three main results were reported. First, we found a differential expression of 10 miRNAs from 754 studied miRNAs between hypertensive patients with and without LVH in an exploratory cohort. Second, we confirmed a differential expression of 5 miRNAs (miR-30a-5p, miR-let7c, miR-92a, miR-451, and miR-145-5p) in an extended sample of hypertensive patients (validation cohort). Of these miRNAs, miR-145-5p, miR-451, and let7-c expression levels were significantly associated with both LVH and LVMI even after adjusting for relevant confounders. Third, results of cell culture assays demonstrated a role of miR-145-5p in the development of cardiac myocyte hypertrophy. These findings provide novel evidence regarding the potential value of previously undisclosed miRNAs as circulating biomarkers or potential mediators of hypertension-induced LV remodeling.

Several studies have suggested that miRNAs play an important role in the LV remodeling and may also serve as circulating biomarkers of LVH (11, 33, 34). However, the knowledge regarding the relationship between circulating miRNAs and LVH in hypertensive individuals is scarce and has been restricted to the evaluation of a small number of pre-selected miRNAs in small samples of patients. In this regard, relationships between LVMI and circulating levels of miR-1, miR-133a, miR-26b, miR-208b, miR-499, and miR-21, miR-9 (12), miR-30e (13), miR-27b (14), miR-29 (15), miR-7, and miR-26 (34) have been reported. Notably, none of these miRNAs showed an association with LVH in our study. These divergences could be explained by differences in sample size, and in ethnic and clinical characteristics among the studied samples, and reinforce the need of confirmatory studies in alternative populations. It is also worth mentioning that the miRNAs which were associated with LVH in our validation cohort were derived from the evaluation of 754 miRNAs in an exploratory sample of 42 hypertensive individuals, thus strengthening the validity of our findings.

In our analysis, there was a significant association of circulating miR-145-5p levels with LVMI and LVH. We then evaluated the impact of this miRNA on cardiac myocyte hypertrophy *in vitro*. The results of cell assays showed that treatment with norepinephrine, a hypertrophic stimulus, increased the expression of miR-145-5p, while overexpression of miR-145-5p also upregulated the expression of markers of cardiac myocyte hypertrophy. In addition, transfection of anti-miR-145-5p markedly decreased norepinephrine-induced expression of cardiac myocyte hypertrophy markers. These data support the notion that miR-145-5p may play a role in the pathophysiology of cardiac myocyte hypertrophy. Conversely, our findings are in contrast with previous *in vitro* studies which showed that miR-145 transfection attenuated isoproterenol- and angiotensin II-induced cardiac myocyte hypertrophy (30, 31) but in agreement with the results of Xu et al. (32) who showed that miR-145 expression increases in induced hypertrophic cardiomyocytes by angiotensin II. The reasons for these discrepancies are not clear, but it is possible that differences in the experimental protocol among the studies might have played a role in this regard. In our protocol, HL-1 cells were treated or not with norepinephrine for 2 weeks, transfected with miR-145-5p or miR-145-5p antagonist, and then analyzed 48 h after. In the study by Lin et al. (30) H9C2 cells were overexpressed with miR-145-5p for 6 h, and then treated with Angiotensin-II for 24 h before analyses, while in the study by Li et al., neonatal rat cardiac myocytes from 1-day-old Sprague–Dawley rats were transfected with miR-145 adenovirus for 48 h, and then stimulated with isoproterenol for 24 h before analyses (31). Regardless of the divergences in the results of *in vitro* studies, our findings provide novel evidence that miR-145-5p may be a circulating marker of LV hypertrophy among hypertensive individuals.

Our data showed that not only miR-145-5p, but also miR-30a-5p, miR-let7c, miR-92a, miR-451 had an independent relationship with LVH in both validation and exploratory cohorts. Notably, these latter 4 miRNAs (miR-30a-5p, miR-let7c, miR-92a, miR-451) have been previously reported to be involved in adverse cardiac remodeling in experimental models (35–38). In addition, we found that only miR-145-5p, miR-let7c, and miR-451 were related to both LVH and LVMI, and therefore could be more specifically involved in the development of hypertension-induced LV hypertrophy. To better understand the biological relevance of these three miRNAs, functional enrichment analysis was performed aiming at identifying their targeted genes and pathways. We found that these miRNAs regulate genes and pathways related to cardiac remodeling and vascular diseases, including metabolic, inflammatory, focal adhesion, calcium and adrenergic signaling pathways. Overall, the current findings suggest that miR-145-5p, miR-let7c, and miR-451 comprise an attractive group of miRNAs that might play a role in hypertension-induced LV remodeling and could emerge as potential biomarkers or therapeutic targets in this regard.

Hypertension is classically assumed to induce LV concentric hypertrophy due to increases in LV wall stress (5). However, several other factors, including hemodynamic variables, as well as acquired and genetic factors may modulate LV geometry in hypertensive individuals, resulting in alternative LV geometric patterns, such as eccentric hypertrophy (5, 39). Available evidence obtained in experimental and clinical settings has also suggested that miRNAs may play a role in the development of LV geometric patterns (11, 40, 41). In our study, we found that patients with eccentric hypertrophy had increased expression of miR-miR-let7c, and miR-145-5p, while those with concentric hypertrophy had higher expression of miR-let7c, miR-92a, miR-145-5p, miR-30a-5p, and miR-451. As far as we know, this is the first study to describe the expression of these aforementioned miRNAs as a function of LV geometric patterns in hypertensive patients and further indicate that the assessment of LV geometry can also be a target in studies focusing on the role of miRNAs in hypertension-induced LV remodeling.

We acknowledge that our study has some limitations. First, we only selected six miRNAs for validation among the miRNAs that were differentially expressed between hypertensive patients with and without LVH in the exploratory cohort. Therefore, further studies should be performed to validate the remaining miRNAs whose expression was different between the two groups. Second, as any cross-sectional study, the influence of residual confounding cannot be excluded and the association between miRNAs and LV structural parameters cannot be assumed to be causal. Third, it is possible that the use of antihypertensive medications and clinical characteristics might have influenced our findings. In order to overcome this limitation, we included each antihypertensive class and potential relevant clinical confounders as independent variables in multivariable analyses regarding the validation cohort. Conversely, because the major aim of the exploratory cohort was to perform miRNA mining and due to its smaller number of enrolled patients, we opted to only perform unadjusted analyses for the relationship of miRNAs with LVH in this cohort.

In conclusion, starting from an analysis of 754 miRNAs, we found that the circulating levels of three miRNAs (miR-145-5p, miR-451, and let7-c) were associated with LVH and left ventricular mass index in hypertensive individuals. Furthermore, *in vitro* studies showed that miR-145-5p may have a pro-hypertrophic effect on cardiac myocytes. These findings suggest that miR-451 and let7c, and especially miR-145-5p, may be potential circulating biomarkers or may be involved hypertension-induced LV remodeling.

## Data Availability Statement

The original contributions presented in the study are included in the article/Supplementary Material, further inquiries can be directed to the corresponding author/s.

## Ethics Statement

The studies involving human participants were reviewed and approved by was approved by the Ethics Committee of the State University of Campinas. 56841616.5.0000.5404. The patients/participants provided their written informed consent to participate in this study.

## Author Contributions

EL, RS, and WN designed this study, acquired and interpreted data, and wrote the manuscript. LP, EOM, LC-R, ERM, CV, JP-M, JM-S, OC-F, and AS acquired and/or interpreted data and revised manuscript for intellectual content. All authors read and agreed with the published version of the manuscript.

## Funding

The study was supported by São Paulo Research Foundation (FAPESP 2017/23563-1) for Dr. Schreiber and by FAPESP 2013/07607-8.

## Conflict of Interest

The authors declare that the research was conducted in the absence of any commercial or financial relationships that could be construed as a potential conflict of interest.

## Publisher's Note

All claims expressed in this article are solely those of the authors and do not necessarily represent those of their affiliated organizations, or those of the publisher, the editors and the reviewers. Any product that may be evaluated in this article, or claim that may be made by its manufacturer, is not guaranteed or endorsed by the publisher.

## Abbreviations

BP: blood pressure
LV: left ventricular
LVM: left ventricular mass
LVMI: left ventricular mass index
LVH: left ventricular hypertrophy
miRNA: microRNA
Nppb: brain natriuretic peptide gene
Nppa: atrial natriuretic peptide gene
RT-qPCR: real-time quantitative polymerase chain reaction.
