# Supplementary material for: Relationship Between Circulating MicroRNAs and Left Ventricular Hypertrophy in Hypertensive Patients
Source: Front Cardiovasc Med. 2022 Apr 13;9:798954. doi: 10.3389/fcvm.2022.798954 (PMC9043518; doi:10.3389/fcvm.2022.798954)
Supplement: Supplementary file 1 [file Data_Sheet_1.docx]

ONLINE SUPPLEMEMT

**Relationship between circulating MicroRNAs and left ventricular hypertrophy in hypertensive patients.**

**Running title:** Cardiac hypertrophy and microRNAs

**Authors:** Elisangela C.P. Lopes; Layde R. Paim; Luís F.R.S. Carvalho-Romano; Edmilson R. Marques; Eduarda O.Z. Minin; Camila F.L. Vegian; José A. Pio-Magalhães; Lício A. Velloso; Otavio R. Coelho-Filho; Andrei C. Sposito; José R. Matos-Souza; Wilson Nadruz Jr; Roberto Schreiber.

Department of Internal Medicine, School of Medical Sciences, State University of Campinas, São Paulo, Brazil.

Address for correspondence:

Roberto Schreiber, Ph.D.

E-Mail: [robertos@unicamp.br](mailto:robertos@unicamp.br).

Wilson Nadruz Junior, MD, Ph.D.

E-Mail: [wilnj@fcm.unicamp.br](mailto:wilnj@fcm.unicamp.br).

Laboratório de Biologia Cardiovascular, Faculdade de Ciências Médicas, Rua Vital Brasil 50- Universidade Estadual de Campinas, Cidade Universitária “Zeferino Vaz”. CEP: 13081-970 Campinas, SP. Brasil.

Phone: (55) (19) 3521 7364

**EXPANDED MATERIAL AND METHODS**

**Study Populations**

The present study included two cohorts of consecutive hypertensive patients followed at the Hypertension Outpatient Clinic of the Clinics Hospital of the University of Campinas who were enrolled from 2018 to 2019. The exploratory cohort included 42 patients (26 with LVH and 16 without LVH) and the validation cohort comprised 297 patients (162 with LVH and 135 without LVH). Exclusion criteria were age under 18 years, identifiable causes of secondary hypertension, evidence of significant cardiac valve disease and hypertrophic cardiomyopathy. The research was carried out in accordance with the Declaration of Helsinki. This study was approved by the Human Research Ethics Committee of the University of Campinas, and all patients gave written informed consent to participate.

**Clinical, Laboratory and Echocardiography Data**

Clinical data were gathered from each participant and included information on: age, sex, smoking, hypertension, diabetes mellitus, use of antihypertensive medications, body mass index (BMI), BP, heart rate. BP and heart rate were measured in the sitting position using a validated digital oscillometric device (HEM-705CP; Omron Healthcare, Kyoto, Japan) with appropriate cuff sizes. BMI was calculated as body weight divided by height squared (kg/m^2^). Fasting low density lipoprotein (LDL)-cholesterol, high-density lipoprotein (HDL)-cholesterol, triglycerides, creatinine, and glucose levels were measured using standard laboratory techniques. Hypertension was defined as systolic BP ≥140 mmHg or diastolic ≥90 mmHg or use of antihypertensive medications. Diabetes mellitus was diagnosed if fasting blood glucose was ≥126 mg/dl or when participants were taking hypoglycemic medications.

Echocardiography was performed by a single physician using a Vivid q device (General Electric, Milwaukee, Wisconsin, USA) equipped with a 3S-RS transducer, as previously described (1-3). LVMI was calculated as LV mass/body surface area. Relative wall thickness (RWT) was measured as 2*posterior wall thickness/LV diastolic diameter. LVH was defined as LVMI ≥95 g/m^2^ and ≥ 115 g/m^2^ in women and men, respectively. LV geometric patterns were defined as follows: normal geometry (No LVH and RWT ≤0.42), concentric remodeling (No LVH and RWT >0.42), eccentric hypertrophy (LVH and RWT ≤0.42) and concentric hypertrophy (LVH and RWT >0.42). LV ejection fraction was estimated by the Simpson's method.

**Extraction and Analysis of Serum miRNA Expression**

For the extraction of serum miRNAs, whole blood samples were collected in tubes containing separator gel and clot activator (BD Vacutainer) and subjected to centrifugation at 2000 rpm for 15 min at room temperature. The top serum layer was immediately aliquoted into RNase-free tubes and immediately frozen at -80°C until RNA extraction. To detect hemolysis in serum samples, we used a spectrophotometer to measure the absorbance of oxyhemoglobin at 414 nm. Serum samples with a reading of 414 >0.2 were considered hemolyzed and excluded.

Samples from both cohorts were extracted using the miRNeasy Serum/Plasma Kit (Qiagen). The quality of miRNA was assessed by measuring the percentage of miRNAs in the amount of small RNA using a Bioanalyzer 2100 (Agilent, Santa Clara, CA), as previously reported (4). In the exploratory cohort, the miRNA profile was analyzed with the TaqMan OpenArray Human MicroRNA system, a quantitative polymerase chain reaction (qPCR)-based miRNA array platform that contains 754 microRNAs on a microfluidic platform across two sets of primer pools, panel A and B (LifeTechnologies). Data were normalized using the global normalization method as suggested by the manufacturer and previous reports (5). Six circulating miRNAs with the highest fold change in the exploratory study were chosen for the validation in the validation cohort by qRT-PCR using a customized plate (LifeTechnologies). Reverse transcription was performed using the SuperScript®III First Strand Synthesis Kit. Specific miRNA PCR primers were synthesized by Life Technologies. Real-time PCR assays were performed with the TaqMan Master Mix (Life Technologies) on a 7900HT FAST Real- Time PCR System (Life Technologies). The comparative Ct (ΔΔCt) method to quantify relative gene expression was used, and fold change (FC) was calculated as FC = 2- ^ΔΔCt^, where Ct is defined as the PCR cycle number at which the fluorescence meets the threshold in the amplification plot (6). Data were normalized using a geometric mean of U6 snRNA (noncoding small nuclear RNA) and miR-16 as the housekeeping genes.

**Gene Set Enrichment Analysis**

To understand the biological relevance of differentially expressed miRNAs, we performed functional enrichment analysis. The miRNAs differentially expressed between patients with and without LVH that significantly correlated with LVMI were uploaded into miRWalk (version 2) (7). To strengthen the data, only mRNAs predicted in at least four of five tools (miRanda, miRDB, miRWalk, RNA22, and TargetScan) were considered as possible miRNA targets. We used the Database for Annotation, Visualization, and Integrated Discovery (DAVID) to determine the enriched pathways. Only pathways with more than 10 genes, fold enrichment >1.5, and Fisher’s exact test p-value <0.05 were considered (8).

**HL-1 Cells Culture and miRNA Transfection**

We used the adult mouse atrial muscle cell line HL-1, which has been extensively used to assess mechanisms related to cardiac myocyte hypertrophy (9,10), to evaluate the functional impact of selected miRNAs. HL-1 cells were cultured at 37°C and 5% CO2 atmosphere in Claycomb medium (Sigma,51800C) supplemented with 2 mM L-Glutamine (Sigma,G7513), penicillin-streptomycin (Sigma,P4333) and 10 %(v/v) FCS (Sigma; F2442) in the absence or presence of 100 µM of norepinephrine (Sigma, A0937) as a hypertrophic stimulus (11,12) for 2 weeks (13) on gelatin-fibronectin [0.02 % (w/v) gelatin; 5 mg/ml fibronectin] (Sigma,F1141) pre-coated plates (14). Then, HL-1 cells were transiently transfected with miR-145-5p mimetic, anti-miR-145-5p or negative control miRNA (Applied Biosystems, ThermoFisher Scientific, Grand Island, NY) at final concentrations of 30 nM. This assay was carried out using the Lipofectamine 2000 Reagent (Invitrogen, Carlsbad, CA) following the manufacturer’s instructions. Cells were harvested for subsequent analyses 48 h after the transfection. All transfection experiments were carried out in triplicate.

**Real-time Quantitative PCR Analysis from Cells Culture**

For detecting the *in vitro* expression of miRNAs and markers of cardiac myocyte hypertrophy, total RNA from cardiac myocytes was isolated using Mirvana Paris Kit (Applied Biosystems, CA). We evaluated the expression of brain natriuretic peptide (Nppb) and atrial natriuretic peptide (Nppa) genes, which are markers of cardiac myocyte hypertrophy, and miR-145-5p using the TaqMan Gene Expression Assays Kit (Applied Biosystems, CA). The polymerase chain reaction was performed in a StepOnePlus ™ System (ThermoFisher) real-time PCR system. Gapdh (Mm99999915_g1) was used as endogenous control for Nppb and Nppa expression, while U6 snRNA was used to normalize miR-145 expression.

**Statistical Analysis**

Continuous variables with normal or non-normal distribution are presented as mean ± standard deviation (SD) or median [25th,75th percentiles]. Differences in continuous variables with normal or non-normal distribution between the studied groups (with and without LVH) were evaluated by unpaired student's t-test and Mann-Whitney U-test, respectively. Chi-square test was used to compare categorical variables. Differences in continuous variables derived from cell assays were evaluated by one-way analysis of variance (ANOVA) followed by the Tukey test. The correlation between echocardiographic variables and log-transformed expression of miRNAs was assessed by the Person’s Method. Multivariable linear regression analysis evaluated the association of log-transformed expression of circulating miRNAs with LVH, LVMI and LV geometric patterns in the validation cohort, adjusting for variables that have been reported to influence LV remodeling: age, sex, diabetes, body mass index, systolic blood pressure, creatinine, smoking and antihypertensive classes (15). p-value <0.05 was considered statistically significant. SPSS 15.0 software was used for statistical analyses.

**SUPPLEMENTAL REFERENCE SECTION**

1. De Rossi G, Matos-Souza JR, Costa e Silva AA, Campos LF, Santos LG, Azevedo ER, Alonso KC, Paim LR, Schreiber R, Gorla JI, et al. Physical activity and improved diastolic function in spinal cord-injured subjects. *Med Sci Sports Exerc.* 2014; 46:887-92.
2. Lacchini R, Jacob-Ferreira ALB, Luizon MR, Coeli FB, Izidoro-Toledo TC, Gasparini S, Ferreira-Sae MC, Schreiber R, Nadruz Jr W, Tanus-Santos JE. Matrix metalloproteinase 9 gene haplotypes affect left ventricular hypertrophy in hypertensive patients. *Clin Chim Acta* 2010; 411:1940-1944.
3. Lacchini R, Jacob-Ferreira ALB, Luizon MR, Gasparini S, Ferreira-Sae MC, Schreiber R, Nadruz Jr W, Tanus-Santos JE. Common matrix metalloproteinase 2 gene haplotypes may modulate left ventricular remodelling in hypertensive patients. *J Hum Hypertens*. 2012;23: 171-177.
4. Li Y, Kowdley KV. Method for microRNA isolation from clinical sérum samples. *Anal Biochem*, 2012;431:69-75.
5. Paim LR, Schreiber R, de Rossi G, Matos-Souza JR, Costa E Silva AA, Calegari DR, Cheng S, Marques FZ, Sposito AC, Gorla JI, et al. Circulating microRNAs, Vascular Risk, and Physical Activity in Spinal Cord-Injured Subjects. *J Neurotrauma*. 2019; 36:845-852.
6. Livak KJ, Schmittgen TD. Analysis of Relative Gene Expression Data Using Real-Time Quantitative PCR and the 2 -∆∆CT Method. *Methods*, 2001;25:402-408.
7. Dweep H, Sticht C, Pandey P, Gretz N. miRWalk—database: prediction of possible miRNA binding sites by “walking” the genes of three genomes. *J Miomed inform.* 2011; 44:839-47.
8. Zhang X, Wang X, Wu J, Peng J, Deng X, Shen Y, Yang C, Yuan J, Zou Y. The diagnostic values of circulating miRNAs for hypertension and bioinformatics analysis. *Biosci Rep*, 2018;38: BSR20180525.
9. White SM, Constantin PE, Claycomb WC. Cardiac physiology at the cellular level: use of cultured HL-1 cardiomyocytes for studies of cardiac muscle cell structure and function. Am J Physiol Heart Circ Physiol. 2004;286:H823-H829.
10. Hao L, Ren M, Rong B, Xie F, Lin MJ, Zhao YC, Yue X, Han WQ, Zhong JQ. TWEAK/Fn14 mediates atrial-derived HL-1 myocytes hypertrophy via JAK2/STAT3 signalling pathway. J Cell Mol Med. 2018; 22:4344-4353.
11. Chen Y, Qiao X, Zhang L, Li X, Liu Q. Apelin-13 regulates angiotensin ii-induced Cx43 downregulation and autophagy via the AMPK/mTOR signaling pathway in HL-1 cells. *Physiol Res*, 2020;69:813-822.
12. Landstrom AP, Kellen CA, Dixit SS, van Oort RJ, Garbino A, Weisleder N, Ma J, Wehrens XH, Ackerman MJ. Junctophilin-2 expression silencing causes cardiocyte hypertrophy and abnormal intracellular calcium handling. Circ Heart Fail. 2011; 4:214-23.
13. Bloch L, Ndongson-Dongmo B, Kusch A, Gragun D, Heller R, Huber O. Real-time monitoring of hypertrophy in HL-1 cardiomyocytes by impedance measurements reveals different modes of growth. *Cytotechnology*, 2016;68:1897-907.
14. Claycomb WC, Lanson Jr NA, Stallworth BS, Egeland DB, Delcarpio JB, Bahinski A, Izzo Jr NJ. HL-1 cells: a cardiac muscle cell line that contracts and retains phenotypic characteristics of the adult cardiomyocyte. *Proc Natl Acad Sci U S A.* 1998; 95:2979-84.
15. Nadruz Jr W. Myocardial remodeling in hypertension. *J Hum Hypertens*. 2015;29: 1-6. doi:10.1038/jhh.2014.36

**Supplementary Tables**

**Table S1.** Clinical, laboratory and echocardiographic characteristics of the cohorts

| Characteristics | Exploratory cohort (n=42) | Validation cohort (n=297) | p |
| --- | --- | --- | --- |
| Age, years | 57.7 ± 8.5 | 61.2 ± 12.2 | 0.062 |
| Male (%) | 22 (52) | 128 (43) | 0.256 |
| Smokers, (%) | 8 (19) | 33 (11) | 0.22 |
| Diabetics, (%) | 17 (40) | 174 (58) | 0.040 |
| Body mass index, kg/m^2^ | 29.5 ± 4.6 | 30.3 ± 5.7 | 0.398 |
| LDL-cholesterol, mg/dL | 113 ± 34 | 93 ± 33 | 0.001 |
| HDL-cholesterol, mg/dL | 48 ± 12 | 46 ± 13 | 0.318 |
| Triglycerides, mg/dL | 118 [90, 208] | 124 [89, 177] | 0.550 |
| Glucose, mg/dL | 100 [89, 129] | 101 [90, 121] | 0.969 |
| Creatinine, mg/dL | 0.90 [0.76, 1.05] | 0.91 [0.75, 1.14] | 0.496 |
| Systolic blood pressure, mm Hg | 140.3 ± 20.1 | 149.0 ± 25.0 | 0.034 |
| Diastolic blood pressure, mm Hg | 76.5 ± 13.2 | 83.5 ± 15.3 | 0.003 |
| Diuretics, n (%) | 32 (76) | 156 (52) | 0.006 |
| CCB, n (%) | 22 (52) | 132 (44) | 0.423 |
| β-Blockers, n (%) | 21 (50) | 129 (43) | 0.524 |
| ACEI or ARB, n (%) | 33 (78) | 261 (88) | 0.155 |
| Interventricular septum thickness, mm | 10.0 ± 1.4 | 10.5 ± 1.8 | 0.047 |
| Posterior wall thickness, mm | 9.9 ± 1.3 | 10.5 ± 1.6 | 0.037 |
| LV end-diastolic diameter, mm | 49.6 ± 4.7 | 49.7 ± 6.1 | 0.953 |
| LV ejection fraction, % | 67.0 ± 5.4 | 65.2 ± 9.5 | 0.297 |
| Relative wall thickness, mm | 0.40±0.05 | 0.43±0.07 | 0.046 |
| LV mass index, g/m^2^ | 120.1 ± 30.9 | 111.5 ± 33.1 | 0.116 |

Continuous data with normal and non-normal distribution are presented as mean ± standard deviation and median [25th, 75th percentiles]. ACEI or ARB – angiotensin-converting enzyme inhibitors or angiotensin receptor blockers; CCB – calcium channel blockers; HDL – high density lipoprotein; LDL – low density lipoprotein; LV – left ventricular.

**Table S2. Differentially expressed miRNAs in participants with left ventricular hypertrophy vs those without left ventricular hypertrophy in exploratory cohort.**

| **miRNA** | **Log2 FC** | **p-value** |
| --- | --- | --- |
|  | **Up-regulated miRNAs** |  |
| **miR-let-7c** | 1.90 | **0.019** |
| **miR-92a** | 1.15 | **0.017** |
| **miR-30a-5p** | 1.15 | **0.023** |
| **miR-145-5p** | 1.49 | **0.030** |
| **miR-451** | 1.08 | **0.027** |
| **miR-375** | 1.05 | **0.018** |
| miR-601 | 1.03 | 0.279 |
| **miR-185** | 0.94 | **<0.001** |
| miR-320B | 0.95 | 0.220 |
| miR-93# | 0.93 | 0.125 |
| miR-10b# | 0.88 | 0.187 |
| miR-1291 | 0.86 | 0.145 |
| miR-30d | 0.84 | 0.129 |
| miR-376c | 0.83 | 0.086 |
| miR-340 | 0.82 | 0.198 |
| miR-328 | 0.79 | 0.114 |
| miR-885-5p | 0.79 | 0.093 |
| **miR-338-5p** | 0.78 | **0.022** |
| miR-122 | 0.78 | 0.064 |
| **miR-10a** | 0.76 | **0.034** |
| miR-484 | 0.74 | 0.155 |
| miR-532-3p | 0.72 | 0.262 |
| miR-193b | 0.72 | 0.107 |
| **miR-296** | 0.68 | **0.034** |
| miR-150 | 0.69 | 0.181 |
| miR-597 | 0.69 | 0.074 |
| miR-423-5p | 0.69 | 0.065 |
| miR-197 | 0.62 | 0.088 |
| miR-766 | 0.62 | 0.278 |
| miR-638 | 0.61 | 0.133 |
| miR-345 | 0.59 | 0.274 |
| miR-99b | 0.57 | 0.153 |
| miR-146a | 0.55 | 0.369 |
| miR-127 | 0.54 | 0.153 |
| miR-193a-5p | 0.54 | 0.155 |
| miR-125b | 0.53 | 0.128 |
| miR-30e-3p | 0.50 | 0.433 |
| miR-194 | 0.50 | 0.221 |
| miR-192 | 0.47 | 0.580 |
| miR-191 | 0.46 | 0.374 |
| miR-660 | 0.44 | 0.137 |
| miR-324-3p | 0.43 | 0.199 |
| miR-29a | 0.41 | 0.647 |
| miR-223# | 0.40 | 0.362 |
| miR-222 | 0.39 | 0.456 |
| miR-365 | 0.39 | 0.482 |
| miR-148b | 0.38 | 0.417 |
| miR-20a | 0.36 | 0.495 |
| miR-21 | 0.36 | 0.260 |
| miR-106b | 0.34 | 0.373 |
| miR-148a | 0.33 | 0.262 |
| miR-19a | 0.32 | 0.549 |
| miR-93 | 0.27 | 0.333 |
| miR-340# | 0.27 | 0.735 |
| miR-126 | 0.26 | 0.596 |
| miR-139-5p | 0.26 | 0.207 |
| miR-18a | 0.24 | 0.393 |
| miR-132 | 0.24 | 0.385 |
| miR-221 | 0.23 | 0.486 |
| miR-19b | 0.23 | 0.576 |
| miR-642 | 0.21 | 0.802 |
| miR-223 | 0.21 | 0.687 |
| miR-181c | 0.20 | 0.684 |
| miR-24 | 0.20 | 0.591 |
| miR-645 | 0.19 | 0.523 |
| miR-144# | 0.19 | 0.731 |
| miR-16 | 0.17 | 0.757 |
| miR-574-3p | 0.16 | 0.714 |
| miR-143 | 0.16 | 0.727 |
| miR-Let-7d | 0.14 | 0.679 |
| miR-106a | 0.13 | 0.661 |
| miR-Let-7g | 0.13 | 0.806 |
| miR-30c | 0.13 | 0.731 |
| miR-532 | 0.12 | 0.748 |
| miR-942 | 0.12 | 0.905 |
| miR-126# | 0.10 | 0.880 |
| miR-17 | 0.10 | 0.732 |
| miR-140 | 0.09 | 0.865 |
| miR-28 | 0.09 | 0.807 |
| miR-125a-5p | 0.09 | 0.865 |
| miR-25 | 0.06 | 0.872 |
| miR-199a-3p | 0.04 | 0.855 |
| miR-146b | 0.03 | 0.952 |
| miR-130b | 0.02 | 0.938 |
| miR-142-3p | 0.02 | 0.953 |
| miR-195 | 0.02 | 0.961 |
| miR-186 | 0.02 | 0.964 |
|  | **Down-regulated miRNAs** |  |
| miR-342-3p | -0.02 | 0.968 |
| miR-323-3p | -0.04 | 0.959 |
| miR-30b | -0.05 | 0.873 |
| miR-101 | -0.06 | 0.917 |
| miR-26a | -0.09 | 0.809 |
| miR-181a | -0.10 | 0.742 |
| miR-20b | -0.13 | 0.712 |
| miR-15b | -0.13 | 0.662 |
| miR-128a | -0.13 | 0.763 |
| miR-548a | -0.14 | 0.863 |
| miR-331 | -0.16 | 0.599 |
| miR-204 | -0.18 | 0.714 |
| miR-28-3p | -0.18 | 0.500 |
| miR-335 | -0.19 | 0.646 |
| miR-103 | -0.20 | 0.432 |
| miR-130a | -0.22 | 0.583 |
| miR-339-3p | -0.22 | 0.693 |
| miR-134 | -0.23 | 0.708 |
| miR-27a | -0.26 | 0.405 |
| miR-361 | -0.26 | 0.477 |
| miR-378 | -0.27 | 0.711 |
| miR-744 | -0.29 | 0.302 |
| miR-376a | -0.31 | 0.805 |
| miR-27b | -0.31 | 0.190 |
| miR-324-5p | -0.33 | 0.317 |
| miR-374-5p | -0.33 | 0.209 |
| miR-26b | -0.38 | 0.143 |
| miR-374 | -0.39 | 0.498 |
| miR-206 | -0.48 | 0.566 |
| miR-27a# | -0.49 | 0.354 |
| miR-151-3p | -0.53 | 0.838 |
| miR-152 | -0.55 | 0.131 |
| miR-301 | -0.61 | 0.076 |
| miR-29c | -1.04 | 0.483 |
| miR-590-5p | -2.74 | 0.200 |

FC- Fold change

**Table S3. Differentially expressed miRNAs in participants with left ventricular hypertrophy vs those without left ventricular hypertrophy in the exploratory and validation cohorts.**

| **miRNA** | Exploratory Cohort | | Validation cohort | |
| --- | --- | --- | --- | --- |
|  | Fold change | p | Fold change | p |
| **miR-Let-7c** | 3.72 | 0.019 | 5.66 | <0.001 |
| **miR-145-5p** | 2.81 | 0.030 | 2.57 | 0.005 |
| **miR-30a-5p** | 2.21 | 0.023 | 1.92 | 0.046 |
| **miR-92a** | 2.22 | 0.017 | 1.45 | 0.041 |
| **miR-185** | 1.92 | <0.001 | -1.27 | 0.358 |
| **miR-451** | 2.11 | 0.027 | 1.34 | 0.032 |
| **miR-375** | 2.07 | 0.018 | - | - |
| **miR-338-5p** | 1.81 | 0.007 | - | - |
| **miR-10a** | 1.69 | 0.034 | - | - |
| **miR-296** | 1.60 | 0.034 | - | - |

**Table S4. Bivariate correlation coefficients between selected miRNAs and diabetes mellitus and systolic blood pressure in the validation cohort.**

| miRNAS | Diabetes mellitus | | Systolic blood pressure | |
| --- | --- | --- | --- | --- |
|  | r | p | r | p |
| Log miR-let7c | -0.051 | 0.197 | 0.037 | 0.547 |
| Log miR-92a | -0.069 | 0.266 | 0.106 | 0.077 |
| Log miR-145-5p | 0.034 | 0.588 | 0.106 | 0.080 |
| Log miR-30a-5p | 0.094 | 0.128 | 0.146 | 0.015 |
| Log miR-451 | 0.134 | 0.057 | 0.213 | 0.002 |
| Log miR-185 | 0.039 | 0.672 | -0.007 | 0.938 |

The correlation of log-transformed expression of miRNAs with systolic blood pressure and diabetes was assessed by the Person’s and Spearman's Method, respectively.

**Table S5. Multivariable linear regression analysis between selected miRNAs and left ventricular mass index or left ventricular hypertrophy in the validation cohort.**

| miRNA | Left ventricular mass index | | Left ventricular hypertrophy | |
| --- | --- | --- | --- | --- |
|  | Beta ± SE | p | Beta ± SE | p |
| Log miR-let7c | 0.015 ± 0.004 | <0.001 | 1.33 ± 0.28 | <0.001 |
| Log miR-92a | 0.006 ± 0.005 | 0.26 | 0.74 ± 0.32 | 0.022 |
| Log miR-145-5p | 0.011 ± 0.004 | 0.005 | 0.89 ± 0.25 | <0.001 |
| Log miR-30a-5p | 0.001 ± 0.003 | 0.74 | 0.54 ± 0.20 | 0.009 |
| Log miR-451 | 0.010 ± 0.004 | 0.019 | 0.79 ± 0.26 | 0.003 |
| Log miR-185 | -0.010 ± 0.007 | 0.12 | -0.04 ± 0.39 | 0.93 |

Legend: All analyses were adjusted for age, sex, body mass index, systolic blood pressure, diabetes mellitus, smoking, creatinine, use of diuretics, beta-blockers, calcium-channel blockers and angiotensin-converting-enzyme inhibitors or angiotensin receptor blockers. Each studied miRNA entered alone in the multivariable regression models. SE – standard error.

**Table S6. Multivariable linear regression analysis evaluating the relationship between miRNA expression and left ventricular geometric patterns in the validation cohort.**

| miRNA | Normal geometry (n=91) | Eccentric LVH (n=53) | Concentric LVH (n=109) | Concentric remodelling (n=44) |
| --- | --- | --- | --- | --- |
| Log miR-let7c | Ref | 1.47 ± 0.42^Ψ^ | 1.48 ± 0.35^Ψ^ | 0.42 ± 0.44 |
| Log miR-92a | Ref | 0.67 ± 0.48 | 1.06 ± 0.40* | 0.52 ± 0.50 |
| Log miR-145-5p | Ref | 1.39 ± 0.37^Ψ^ | 0.62 ± 0.30* | -0.05 ± 0.38 |
| Log miR-30a-5p | Ref | 0.50 ± 0.30 | 0.65 ± 0.25* | 0.18 ± 0.31 |
| Log miR-451 | Ref | 0.75 ± 0.41 | 0.99 ± 0.32^#^ | 0.34 ± 0.39 |
| Log miR-185 | Ref | -1.25 ± 0.65 | 0.05 ± 0.50 | -0.58 ± 0.60 |

Legend: Data are presented as beta coefficient ± standard error. All analyses were adjusted for age, sex, body mass index, systolic blood pressure, diabetes mellitus, smoking, creatinine, and use of diuretics, beta-blockers, angiotensin-converting enzyme or angiotensin receptor blocker and calcium-channel blocker. *p<0.05; #p<0.01; and Ψp<0.001 compared with normal geometry. LVH – left ventricular hypertrophy. Ref- Reference.


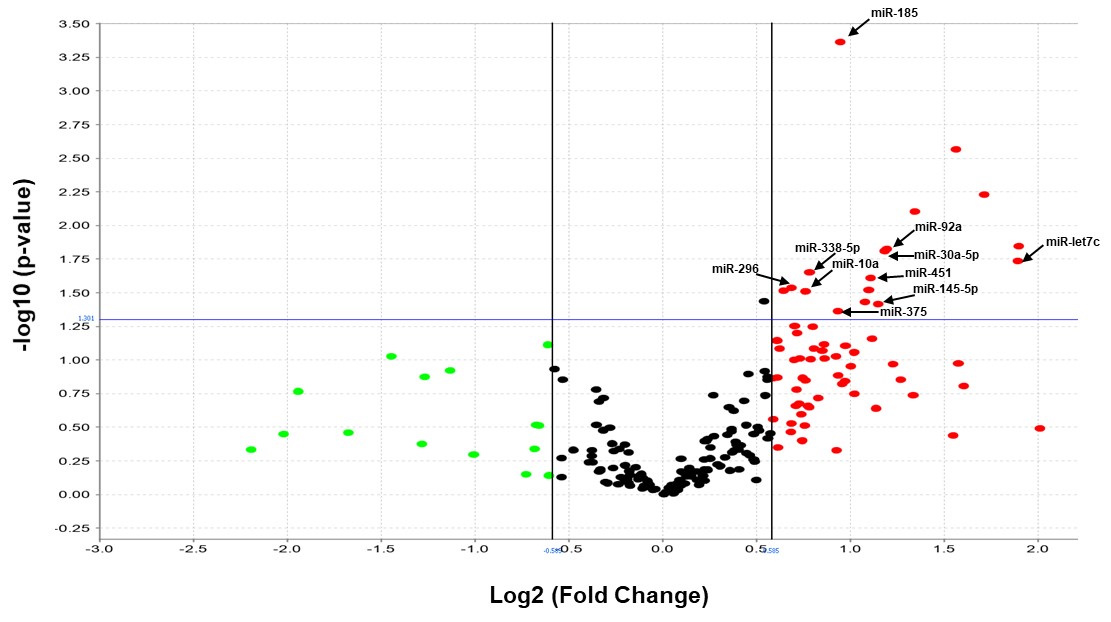


**Figure S1**. Differential expression analysis of microRNAs (miRNAs) obtained in serum of hypertensive patients with left ventricular hypertrophy (LVH) vs without LVH. Volcano plot depicts the significantly altered miRNAs found (p<0.05). Each dot represents a miRNA, upregulated are in red (log2 fold change ≥ 1.5), downregulated in green (log2 fold-change ≤ 1.5), and unchanged in black.
